# Aberrantly higher functional connectivity in the salience network is associated with transient global amnesia

**DOI:** 10.1038/s41598-021-97842-y

**Published:** 2021-10-18

**Authors:** Geon Ha Kim, Bori R. Kim, Min Young Chun, Kee Duk Park, Soo Mee Lim, Jee Hyang Jeong

**Affiliations:** 1 Department of Neurology, College of Medicine, Ewha Womans University Mokdong Hospital, Ewha Womans University, Seoul, Republic of Korea; 2 Ewha Medical Research Institute, Ewha Womans University, Seoul, Republic of Korea; 3 Department of Radiology, Ewha Womans University Seoul Hospital, Ewha Womans University College of Medicine, Seoul, Republic of Korea; 4 Department of Neurology, Ewha Womans University Seoul Hospital, Ewha Womans University College of Medicine, 1071, 260, Gonghang-daero, Gangseo-gu, Seoul, 07804 Republic of Korea

**Keywords:** Diseases, Neurology, Pathogenesis

## Abstract

Triple intrinsic brain networks including the salience network (SN), default mode network (DMN), and central executive network (CEN), are known to be important in human cognition. Therefore, investigating those intrinsic brain networks in transient global amnesia (TGA) may offer novel insight useful for the pathophysiology of TGA. Fifty TGA patients underwent the resting state functional magnetic resonance imaging (rsfMRI) within 24 h, at 72 h, and 3 months after TGA onset. Twenty-five age, gender matched controls also underwent rsfMRI. Within 24 h of TGA onset, TGA patients showed greater functional connectivity in the SN and lower functional connectivity in the DMN, while relatively preserved functional connectivity was observed in the CEN. Interestingly, TGA patients continued to show decreased connectivity in the DMN, while no alterations were shown in the SN 72 h after illness onset. Three months after TGA onset, alterations of functional connectivity in the SN or the DMN were normalized. Our findings suggest that TGA is associated with transient greater functional connectivity in the SN and lower connectivity in the DMN.

## Introduction

Transient global amnesia (TGA) is a clinical syndrome characterized by the sudden onset of anterograde and retrograde amnesia that lasts up to 24 h^1^. Although TGA patients exhibit anterograde amnesia for episodic memory, other memories, including semantic, procedural, and recognition memory, remain intact^2^. In addition, TGA patients do not exhibit focal neurological or other cognitive deficits such as attention. Several factors have been implicated in the pathophysiology of TGA, including migraines, focal ischemia, venous flow abnormalities, and epilepsy^3^. However, the etiology of TGA remains poorly understood.

Previous neuroimaging studies using diffusion-weighted imaging (DWI) have shown that approximately 70% of TGA patients exhibit focal hippocampal lesions in the cornus ammonis (CA)1^4,5^. These hippocampal lesions developed between 24 and 48 h following the onset of TGA^3,6^. However, in a substantial number of patients with TGA, there were no visible DWI lesions of hippocamps even though they had typical symptoms of TGA^3,6^, which might suggest a threshold-dependent phenomenon of TGA that leads to functional deficits of the brain but does not lead to detectable signal changes on magnetic resonance imaging (MRI).

Recent advances in functional neuroimaging have permitted the investigation of large-scale functional brain networks. Previous research has suggested that the triple major brain network models are related to cognition^7–10^, which include the salience network (SN), default mode network (DMN), and central executive network (CEN)^11^. It has been noted that the SN and CEN typically demonstrate increased connectivity in response to external salient events or tasks^11,12^, while the DMN is associated with episodic memory retrieval and autobiographical memory^11,13,14^. Previous research suggested that abnormal organization and functioning of those triple neurocognitive networks are prominent features of several neuropsychiatric disorders^11^.

Although there have been several functional neuroimaging studies of TGA, most studies have focused on the hippocampus and hippocampal networks^15,16^. Recent previous study with resting-state functional MRI (rsfMRI) in TGA patients has shown that decreased functional connectivity on memory network including hippocampus, parahippocampal gyrus and posterior cingulate gyrus, which is more prominent in the hyperacute phase than the post-acute phase^16^. In addition, more recent study with resting state connectivity reported that TGA patients showed reduced functional connectivity in executive network such as frontal, parietal and insular cortex^17^. However, there were few studies that investigated alterations of triple intrinsic brain network in TGA patients. Given that up to 90% of TGA patients reported precipitating causes such as physical or emotional stress prior to the amnestic symptoms^18^, investigating intrinsic brain networks may offer novel insight that may be useful for examining the pathophysiology of TGA.

The aim of this study was to investigate whether TGA patients have alterations in the triple intrinsic brain networks in the SN, DMN, and CEN within 24 h as well as at 72 h and 3 months after symptom onset.

## Results

### Demographic characteristic

There were no significant demographic differences between the TGA and control groups regarding age, sex, and years of education (Table 1). There were no significant group differences in vascular risk factors such as hypertension, diabetes, cardiac disease, and history of stroke. TGA patients had more migraines compared to the controls; however, this was not statistically significant (*P* = 0.05).

**Table 1:** Demographic characteristics.

Mean ± SD	Controls (n = 25)	TGA (n = 50)			*P for group* (Controls vs Total TGA)
			Hyperacute (n = 26)	Post-acute (n = 24)	
Age (years)	60.2 ± 5.8	60.6 ± 6.3	60.8 ± 6.8	60.4 ± 5.8	0.76
Education (years)	9.8 ± 4.6	11.2 ± 3.6	10.2 ± 3.3	12.3 ± 3.7	0.16
Female, n (%)	19 (76.0)	42 (84.0)	22 (84.6)	20 (83.3)	0.40
***Risk factors***					
Hypertension, n (%)	4 (16.0)	14 (28.0)	5 (19.2)	9 (37.5)	0.25
Diabetes mellitus, n (%)	4 (16)	3 (6.0)	2 (7.7)	1 (4.2)	0.16
Hyperlipidemia, n (%)	3 (12)	10(20.0)	4 (15.4)	6 (25)	0.39
Cardiac disease, n (%)	2 (8.0)	4 (8.0)	1 (3.9)	3 (12.5)	1.0
Stroke, n (%)	0 (0)	3(6.0%)	0 (0)	3 (12.5)	0.21
Migraine, n (%)	0 (0)	7(14%)	5 (19.2)	2 (8.3)	0.05
***TGA symptoms***					
Duration of TGA symptoms (min)	NA	497.8 ± 313.5	513.0 ± 311.6	481.3 ± 321.7	NA
Time lapse from symptom onset to MRI scan (min)	NA	689.6 ± 425.0	420.8 ± 231.1	1137.5 ± 260.3	NA
K-MMSE (during ER visit)	28.6 ± 1.4	23.1 ± 3.2	22.8 ± 3.3	23.4 ± 3.2	< 0.001*
Time orientation	4.9 ± 0.3	2.3 ± 1.6	2.2 ± 1.5	2.4 ± 1.8	< 0.001*
Place orientation	5.0 ± 0.2	4.3 ± 0.8	4.2 ± 0.8	4.4 ± 0.7	< 0.001*
Registration	3.0 ± 0	3.0 ± 0	3.0 ± 0	3.0 ± 0	**NA**
Attention and calculation	4.5 ± 0.6	4.0 ± 1.1	4.2 ± 1.1	3.8 ± 1.2	**NA**
Recall	2.4 ± 0.9	0.9 ± 1.0	0.9 ± 1.0	1.1 ± 0.9	< 0.001*
Language	7.9 ± 0.3	7.7 ± 0.5	7.6 ± 0.5	7.7 ± 0.5	**0.02**
Interlocking pentagon drawing test	1.0 ± 0	1.0 ± 0.2	1.0 ± 0	0.9 ± 0.2	**0.43**
K-MMSE (72 h)	NA	28.8 ± 1.2	28.5 ± 1.2	29.0 ± 1.2	NA
K-MMSE (3 months)^a^	NA	29.5 ± 1.0	29.2 ± 1.3	29.8 ± 0.4	NA

^a^Only 22 TGA patients were evaluated at 3 months after onset.

*K-MMSE* Korean version of the Mini Mental State Examination, *SD* standard deviation, *TGA* transient global amnesia, *NA* not applicable, *ER* emergency room.

The mean duration of TGA symptoms was 497.8 ± 313.5 min, and the mean time elapsed between the onset of TGA and the baseline MRI scan was 689.6 ± 425.0 min. Among 50 TGA patients, 26 patients were regarded as hyperacute phase, which referred to those who still had TGA symptoms such as temporal disorientation or repetitive questioning during the baseline MRI scan, whereas 24 TGA patients were regarded as the post-acute phase that referred to TGA patients who no longer exhibited repetitive questioning but still displayed memory deficits on MMSE. TGA patients had lower mean K-MMSE scores during the acute phase (22.7 ± 3.3) compared to follow-up visits, including at 72 h after illness onset (28.6 ± 1.4) and 3 months after illness onset (29.5 ± 1.0).

**Figure 1:**
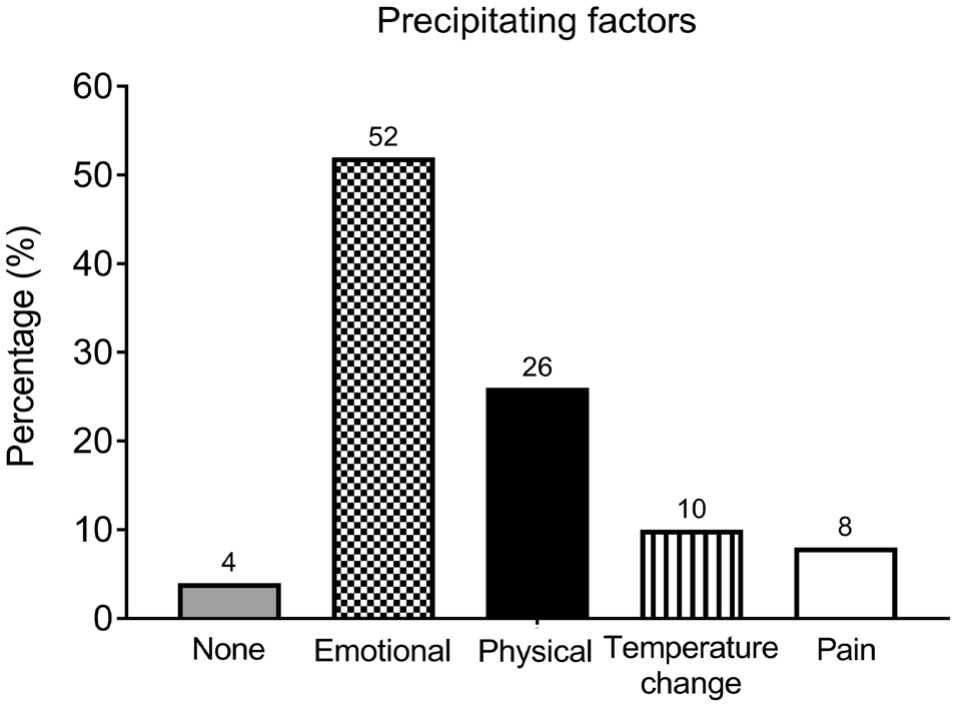
Percentage of precipitating factors in TGA patients. Emotional stress was reported by 52% of TGA patients. Emotional stress included arguments or physical fights with others and the death of a relative or friend. Physical activity, including heavy lifting, climbing, cycling, and swimming, precipitated 26% of TGA cases.

Emotional stress, including arguments with others and death of relatives or friends, precipitated 52% of TGA cases. Physical activity, including heavy lifting, climbing, and swimming, precipitated 26% of TGA cases (Fig. 1).

All 50 TGA patients with acute phase participated in the follow-up MRI 72 h after illness onset, while only 22 patients participated in the 3-month follow-up MRI scan. Among 50 patients with TGA who visited to ER, 34 (68.0%) had a hyperintense lesion on the hippocampus according to DWI during the MRI examination at 72 h after illness onset. Clinical characteristics of participants who participated at 3-month follow up visit (n = 22) and of those who did not (n = 28) are shown in Table e-1.

### Primary analyses

#### Alterations in functional networks during the acute phase of TGA

**Figure 2:**
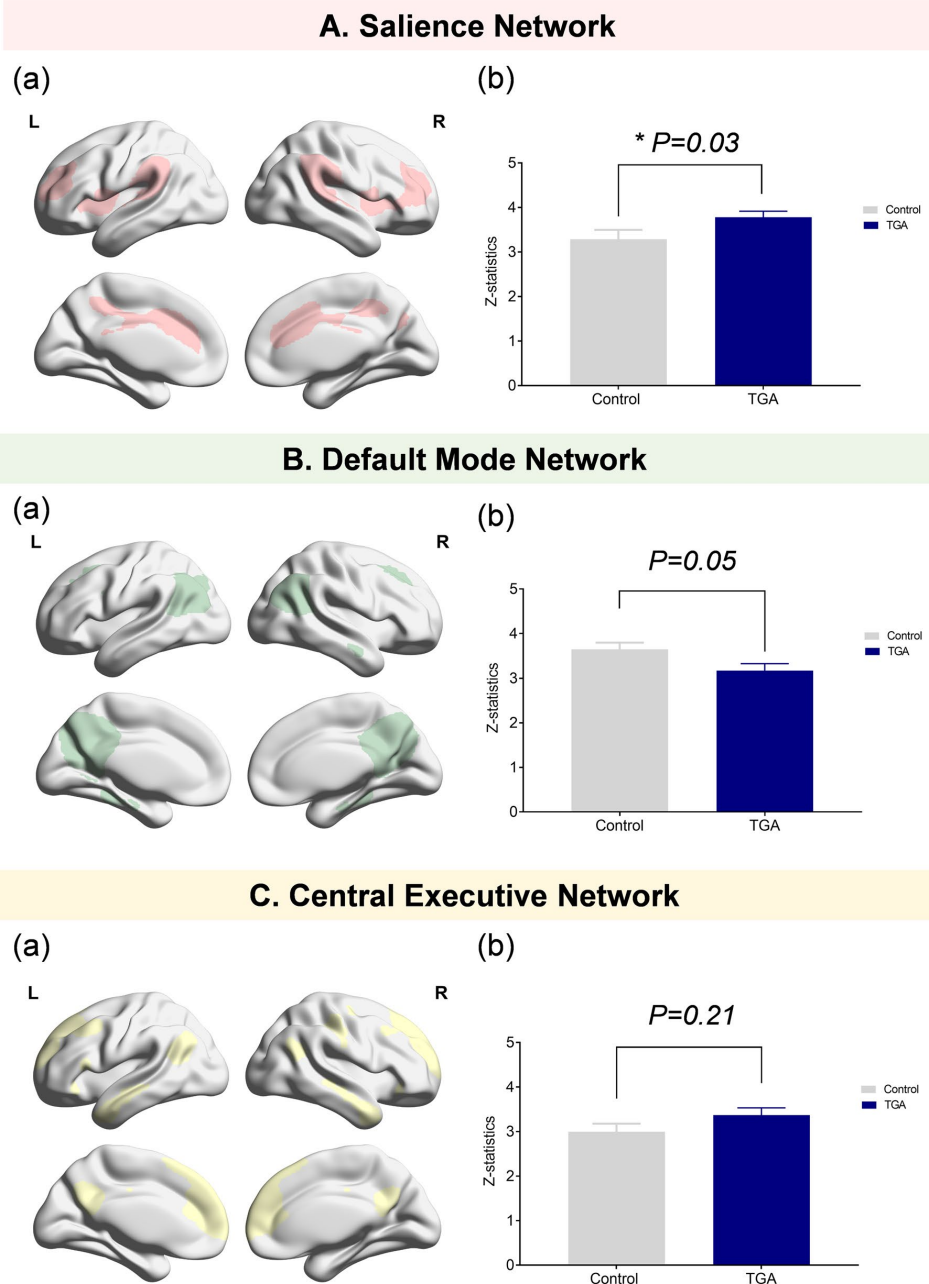
Group differences in the functional intrinsic brain network during the acute phase. A (**a**) Template for the salience network (SN). (**b**) Compared to controls, TGA patients during the acute phase of the illness showed higher functional connectivity in the SN. B (**a**) Template for the default mode network (DMN). (**b**) Compared to controls, TGA patients demonstrated lower functional connectivity in the DMN during the acute phase, but the statistical significance was equivocal. C (**a**) Template for the Central Executive Network (CEN). (**b**) There were no significant group difference of functional connectivity on the CEN between TGA patients and controls. *P value was adjusted by age and sex.

Within 24 h of illness onset, TGA patients showed greater functional connectivity in the SN but relatively lower functional connectivity in the DMN compared to controls (Fig. 2) after adjusting age and sex.

**Figure 3:**
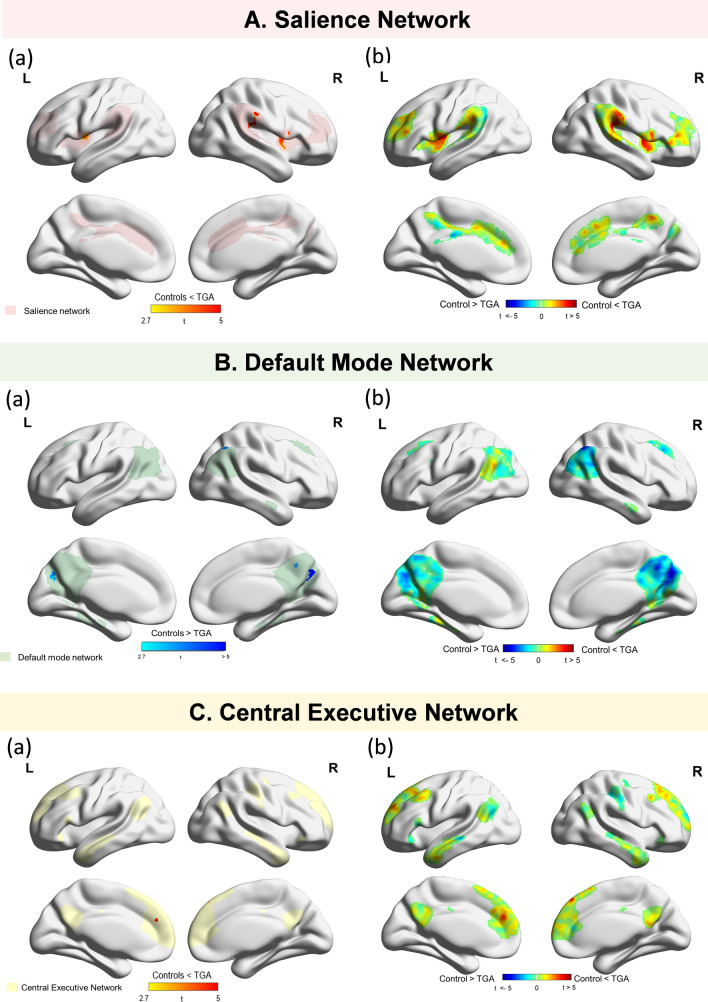
Topographical differences in the functional intrinsic brain network of controls and TGA patients during the acute phase. A (**a**) Significant clusters indicating the group differences of functional connectivity in the SN. Compared to controls, TGA patients showed greater functional connectivity in the bilateral insular cortices and operculum within the SN. There were no significant areas that demonstrated greater functional connectivity in the controls within the SN. (**b**) T-static maps of functional connectivity for the group differences in the SN of TGA patients and controls. B (**a**) Significant clusters indicating the group differences in functional connectivity in the DMN. TGA patients showed lower functional connectivity in the bilateral precuneus within the DMN compared to controls. However, there were no significant areas that showed lower functional connectivity in the controls within the DMN. (**b**) T-static maps of functional connectivity for the group differences in the DMN in TGA patients and controls. C (**a**) Significant clusters indicating the group differences of functional connectivity in the CEN. Topographical analysis illustrated that higher functional connectivity of the TGA patients was observed on the small areas of the medial prefrontal cortex within the CEN compared to that of the controls. (**b**) T-static maps of functional connectivity for the group differences in the CEN of TGA patients and controls.

The topographical analysis demonstrated that TGA patients with acute phase showed greater functional connectivity, especially in the bilateral insular cortex and operculum, within the SN, but had lower functional connectivity in the bilateral precuneus within the DMN (Fig. 3, Table 2). Within 24 h of illness onset, the global functional connectivity in the CEN was not statistically different between TGA patients and controls, although a topographical analysis illustrated that higher functional connectivity of the TGA patients was observed on the small areas of the medial prefrontal cortex within the CEN compared to that of the controls.

**Table 2:** Cluster information of different functional connectivity between the controls and the TGA patients.

	Cluster size (mm^3^)	Maximum *t* value	MNI atlas coordinates		
			(location of maximum *t*-value)		
			***x***	***y***	***z***
*Salience Network* (↑ in TGA)					
Fronto-parietal operculum (L)	2424	4.86	48	− 28	20
Anterior insular (R)	2032	4.55	38	0	10
Anterior insular (L)	896	4.5	− 36	− 4	8
Frontal operculum (L)	744	4.11	− 52	− 4	12
Superior frontal (L)	360	3.86	− 62	− 26	20
*Default Mode Network* (↓ in TGA)					
Precunues(R)	1320	4.87	16	− 70	38
Angular gyrus(R)	728	3.71	44	− 60	44
Precuneus(L)	496	3.61	− 14	− 72	− 28
Precunues(R)	440	3.82	8	− 54	42
*Central Executive Network* (↑ in TGA)					
Medial Prefrontal (L)	336	3.63	− 4	44	26

Significant difference of clusters in the default mode, salience and central executive network between the controls and TGA patients. Regions of decreased or increased connectivity in the TGA group were shown at the cluster-corrected *P* < 0.05.

**Figure 4:**
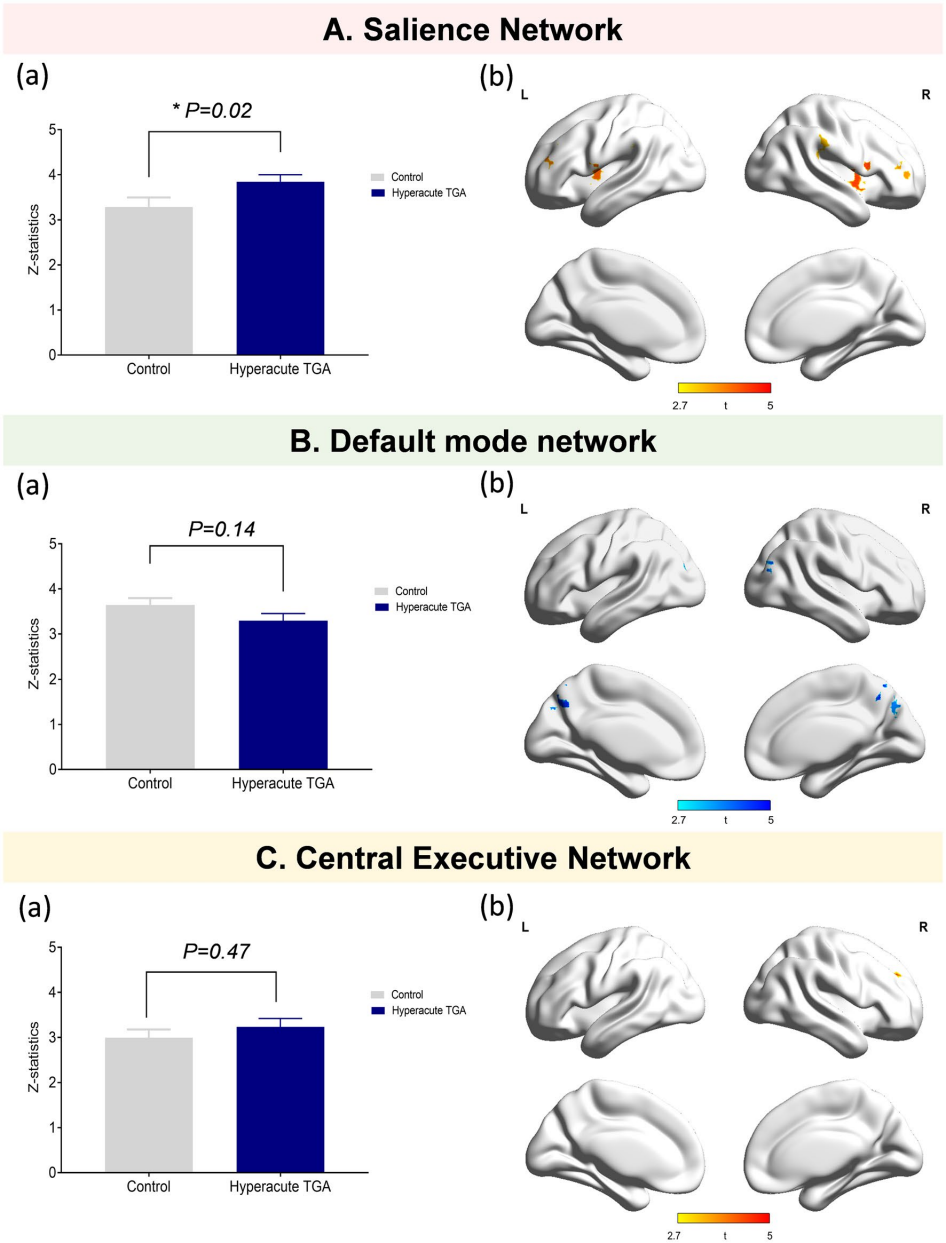
Global and topographical differences in the functional intrinsic brain network between the controls and hyperacute TGA patients. A (**a**) Compared to controls, TGA patients during the hyperacute phase showed higher functional connectivity in the SN. (**b**) Compared to controls, TGA patients showed greater functional connectivity in the bilateral insular and frontal cortices within the SN. B (**a**) There were no significant group difference of functional connectivity on the DMN between hyperacute TGA patients and controls. (**b**) TGA patients with hyperacute phase showed lower functional connectivity in the bilateral precuneus and right inferior parietal cortex within the DMN compared to controls. C (**a**)There were no significant group difference of functional connectivity on the CEN between hyperacute TGA patients and controls. (**b**) Topographical analysis illustrated that higher functional connectivity of the TGA patients with hyperacute phase was observed on the small areas of the right dorsolateral prefrontal cortex within the CEN compared to that of the controls.

Sub-analyses for comparison of functional connectivity between hyperacute TGA patients and controls (Fig. 4) showed that hyperacute TGA patients showed increased functional connectivity in the SN, especially on the bilateral insular cortex, which is comparable to that from whole TGA patients. The topographical analysis also demonstrated that hyperacute TGA patients had lower functional connectivity in the bilateral precuneus within the DMN and greater functional connectivity on the right frontal cortex within the CEN, which is consistent with that from whole TGA patients. However, there were no significant differences of functional connectivity in the SN, DMN and the CEN between the post-acute TGA patients and controls.

#### Longitudinal changes of functional networks in TGA patients

**Figure 5:**
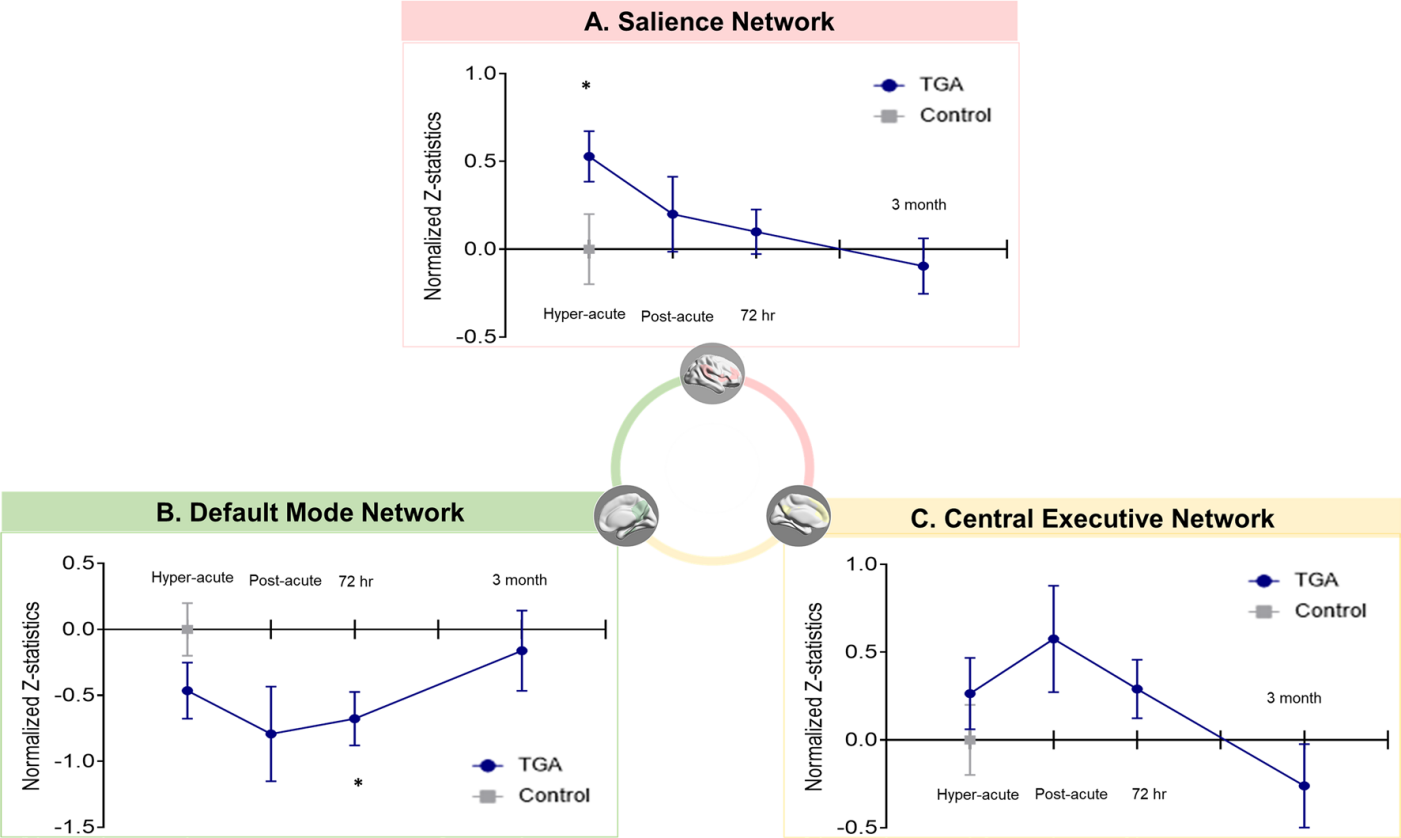
Longitudinal changes of functional connectivity in the intrinsic brain network among TGA patients. (**A**) TGA patients with hyperacute phase showed greater functional connectivity in the SN whereas, those with post-acute phase showed no statistical different functional connectivity in the SN compared to controls. TGA patients did not show greater functional connectivity in the SN at 72 h after TGA onset. Additionally, alterations in functional connectivity in the SN were no longer observed at 3 months after TGA onset. (**B**) Compared to controls, there were no significant differences of DMN in TGA patients with hyperacute phase or in those with post-acute phase. TGA patients continue to show decreased functional connectivity in the DMN 72 h following TGA onset, whereas alterations in functional connectivity in the DMN were no longer shown 3 months following TGA onset. (**C**) Functional connectivity in the CEN was relatively preserved within 24 h of illness onset as well as at 72 h and 3 months after TGA onset.

TGA patients did not show greater functional connectivity in the SN 72 h after TGA onset, while they continued to show decreased functional connectivity in the DMN 72 h after illness onset (Fig. 5). However, alterations in functional connectivity in the SN and DMN were no longer observed at 3 months after TGA onset. Functional connectivity in the CEN was relatively preserved within 24 h of illness onset as well as at 72 h and 3 months after TGA onset. Longitudinal changes of topographical functional connectivity within TGA patients were demonstrated in Figure e-1.

### Secondary analyses

#### Comparison of functional connectivity between TGA patients with hyperacute and those with post-acute phase

When we compared global functional connectivity of the SN, DMN and CEN between TGA patients with hyperacute phase and post-acute phase, there were no significant statistical differences of intrinsic brain network between those two groups (Figure e-2).

#### Comparison of functional connectivity between TGA patients with hippocampal lesion and those without hippocampal lesion on DWI

There were no significant differences of global functional connectivity in the SN, DMN and CEN between TGA patients with hippocampal lesion (n = 34) and those without hippocampal lesion (n = 16) (Figure e-3).

#### Comparison of functional connectivity between TGA patients with emotional cause and those with other causes

To investigate the effects of precipitating emotional causes on intrinsic brain networks, we compared functional connectivity of the SN, DMN and CEN between TGA patients with emotional causes (n = 26) and those with other causes (n = 24). However there were no statistical differences of functional connectivity between the two groups (Figure e-4).

## Discussion

Our main finding was that the TGA patients showed greater functional connectivity in the SN compared to controls in the acute phase, which was normalized in 72 h and 3 months after TGA onset.

In particular, TGA patients with acute phase showed greater functional connectivity in the SN, especially in the bilateral insular cortex. It is well-known that the SN has a role in identifying and integrating interoceptive, autonomic, and emotional information, which leads to detection of the most homeostatically relevant external stimuli to guide behavior^9,12,19^. Therefore, one of the key roles of the SN is switching of the brain networks, leading to the engagement of the CEN and disengagement of the DMN to external stimuli, or the engagement of the DMN and disengagement of the CEN to internal stimuli^9,11^.

Considering that 96% of TGA patients reported the precipitating events such as pain, emotional stress or physical distress prior to clinical amnestic symptoms, aberrantly greater functional connectivity of the SN to external salient stimuli might lead to aberrant disengagement of DMN and engagement of the CEN in TGA patients.

In agreement with this hypothesis, patients with TGA had lower functional connectivity in the DMN, especially in the bilateral precuneus. Because the DMN underlies autobiographical and episodic memory^20–23^, our findings are consistent with the previous studies demonstrating lower functional connectivity in the posterior memory network, including the precuneus in TGA patients^15,16^.

In addition, TGA patients continued to show lower functional connectivity in the DMN at 72 h after illness onset. This finding is consistent with the previous work that showed disruptions in memory networks can be observed between 24 and 48 h after TGA onset^3,6^. One explanation for delayed abnormalities in the DMN could be that TGA patients might have difficulties with rapid allocation of resources from external salient stimuli in the SN to internal mental process in the DMN^10^. Therefore, overactive SN in TGA patients may cause delayed allocation of resources to internal stimuli for TGA patients, which may cause sustained abnormally lower activities in the DMN for TGA patients. However, further studies are warranted to examine whether alterations in these networks are linked to delayed processing of the brain network.

Unexpectedly, compared to controls, TGA patients did not demonstrate aberrant functional connectivity in the CEN, although the focal areas of increased functional connectivity in the medial prefrontal cortex were noted during the acute phase. Previous studies have shown that TGA patients had preserved working memory and executive functions but prominent impaired episodic impairment during an acute attack of TGA^24,25^, therefore, relatively spared functional connectivity of the CEN during acute phase could explain the preserved cognitive function in working memory and executive function during acute phase of TGA. However, because we did not perform neuropsychological tests during the acute phase of TGA in the current study, further studies involving detailed neuropsychological tests during the acute phase of TGA could help reveal the relationship between preserved CEN and executive function in TGA patients.

It should be noted that previous research with intrinsic brain network of TGA showed lower functional connectivity in the executive network during acute phase^17^, which is contrary to our findings. One of possible explanation for this could be that the current study excluded TGA patients with prior history of any psychiatric illness, whereas 31.3% of TGA patient in the previous study had a history of psychiatric illness. The lower functional connectivity of the CEN has been reported in patients with psychiatric diseases ^11,26^, which might affect the different results.

Interestingly, there were no significant differences of functional connectivity of SN, DMN and CEN between the TGA patients with hyperacute and those with post-acute phase (Figs. 1, 2). However, compared to controls, enhanced functional connectivity of the SN was prominent only in the hyperacute phase, whereas a tendency of lower functional connectivity in the DMN was observed only in the post-acute phase (*P* = 0.06) (Fig. 5). This finding can also support that alterations in functional connectivity of the SN might precede alterations in the DMN in TGA patients, as mentioned above.

As expected, alterations of global functional connectivity in the SN and DMN were normalized at the time of the 3-month follow-up examination. This finding is consistent with those of many previous studies that reported that functional changes observed during the acute phase of TGA were normalized at follow-up visits^16,27^. It is also noteworthy that compared to the baseline, TGA patients showed decreased functional connectivity on the right precuneus of the SN and on the left parahippocampal gyrus of the DMN in 72 h. Considering that such areas of decreased functional connectivity in 72 h are involved the DMN, it could be also compatible with our findings illustrating that decreased functional connectivity in the DMN was most prominent in 72 h compared to controls.

There were several limitations to our study. First, we did not include healthy volunteers at the 72 h and 3-month time point. Instead, we compared patients' data from the 72 h and 3-month follow-up visit to the data acquired from healthy controls at the baseline. Second, although there were no significant differences of the baseline clinical characteristics between TGA patients who completed all three follow-up visits compared to those who only participated in two visits, only 22 of 50 patients participated in the 3-month follow-up visit. Third, as mentioned, because we did not perform neuropsychological tests during TGA attacks in the current study, the relationship between alterations in the functional brain network and neuropsychological performance of TGA patients was not explored in this study.

Fourth, it has been known that enhanced SN functional connectivity has been noted in several psychiatric disease such as acute stress, anxiety and post-traumatic stress disorders^11,28,29^. Since 52% of TGA patients in the current study have reported external salient provoking events such as emotional stress, we cannot totally exclude the possibility that increased functional connectivity in the SN in TGA patients might be related to acute stress reaction. However, when we compared the functional connectivity between TGA patients with emotional stress and those with other causes, there were no significant differences of functional connectivity in the SN between those two groups. Furthermore, the functional connectivity of the DMN, one of well-known vulnerable brain network to acute stress^30^, was not different between the two groups either. It may suggest that such alterations of the functional connectivity in TGA patients might represent characteristic alterations of the intrinsic brain network in TGA patients, not mere reactive alterations to acute stress. However, further studies related to functional connectivity according to various precipitating factors could be helpful to elucidate whether a variety of precipitating factors might affect the pathophysiology of TGA differently.

Nonetheless, this is the first study to investigate the intrinsic functional connectivity of TGA patients longitudinally. Our study suggests that greater functional connectivity in the SN and lower functional connectivity in the DMN may be associated with TGA, which are normalized 3 months after illness onset.

## Methods

### TGA patients

TGA patients were prospectively recruited from the Ewha Womans University Mokdong Hospital between March 2015 and October 2017. A diagnosis of TGA was based on the following inclusion criteria^31,32^: the presence of anterograde amnesia, which was witnessed by another individual; no clouding of consciousness or loss of personal identity; the presence of cognitive impairment limited to amnesia; no evidence of neurological abnormalities or epilepsy; no recent history of head trauma or seizures; resolution of amnesia symptoms within 24 h of onset. Exclusion criteria were as follows: suspected or diagnosed mild cognitive impairment or Alzheimer’s disease; severe, major medical, or neurological illnesses; axis I mental illnesses, including current major depressive disorder and lifetime substance use disorders; history of head trauma leading to a loss of consciousness or seizure; any contraindications to MRI; and the use of medications that influence the central nervous system within the past 3 months.

Of 58 TGA patients who visited the emergency department (ER) at the Ewha Womans University Mokdong Hospital, 50 patients agreed to participate in the study. The 6 TGA patients refused to take MRI while 2 patients were excluded due to a history of depression. The 50 TGA patients with acute phase were subdivided into the two groups; hyperacute vs post-acute phase. TGA patients with hyperacute phase referred to those who still had TGA symptoms such as temporal disorientation or repetitive questioning during the baseline MRI scan (n = 26), whereas those with post-acute phase refers TGA patients who no longer exhibited repetitive questioning but still displayed memory deficits on MMSE (n = 24).

TGA patients underwent MRI and clinical assessment using the Korean version of the Mini Mental Status Examination (K-MMSE)^33^ at ER, within 72 h and 3 months following TGA onset. All TGA patients were assessed using electrocardiography (ECG), electroencephalography (EEG), and routine laboratory blood tests following admission. All TGA patients underwent a standard cardiovascular examination involving a structured interview to assess vascular and non-vascular risk factors for TGA (hypertension, diabetes, hyperlipidemia, alcohol use, and smoking). A history of cardiovascular, neurological, and psychiatric diseases was recorded (previous cerebrovascular events, TGA, cardiac disease, migraine, epilepsy, psychiatric disease).

### Controls

We also included 25 cognitively normal individuals matched according to age and sex without any history of TGA (hereafter referred as “controls”). These controls were spouses of outpatients visiting the Memory Disorder Clinic at Ewha Womans University Mokdong Hospital during the same period and who did not have a history of or current TGA or other neurological or psychiatric illnesses. Controls were selected according to their K-MMSE results^33^ and Seoul Neuropsychological Screening Battery results^34^. The SNSB is an extended neuropsychological battery that includes tests measuring attention, language, calculation, praxis, visuospatial abilities, verbal/visual memory, and executive functioning. Normal cognitive function was defined when individuals scored 1 standard deviation (SD) or more below the population norm on the memory, attention, language, visuospatial, and frontal tests of the SNSB.

### Standard protocol approvals, registrations, and patient consents

Written informed consent prior to study participation was obtained from all participants or their legally authorized representatives in accordance with the Declaration of Helsinki., which was repeated in acute TGA patients after symptom resolution. The Institutional Review Board of Ewha Womans University Mokdong Hospital examined and approved the study protocol (EUMC 2015-07-003-016).

### Functional magnetic resonance imaging acquisition and analysis

A 3-Tesla Philips Achieva MR scanner (Philips Medical Systems, Noord-Brabant, Netherlands) was used to acquire MR images. The routine MRI protocol for TGA consisted of T1-weighted and T2-weighted imaging, fluid-attenuated inversion recovery, and conventional gradient-echo images in the transverse plane and three-dimensional (3D) time-of-flight angiography of the intracranial region. Diffusion-weighted imaging (DWI) was performed at 72 h after onset to detect high signal intensities on the hippocampus of TGA patients. DWI was performed in the transverse and coronal planes covering the entire brain with b = 1000 (s/mm^2^) and 3-mm slice thickness.

For functional network analysis, structural images were acquired using a 3D T1-weighted magnetization-prepared rapid gradient-echo imaging sequence with the following acquisition parameters: repetition time (TR), 9.9 ms; echo time (TE), 4.6 ms; flip angle (FA), 8º; field of view (FOV), 240 × 240 mm^2^; slice thickness, 1 mm; number of excitations (NEX), 1; number of slices, 160; and orientation, sagittal. Resting state functional images were acquired using an echo planar imaging sequence with the following parameters: TR, 3,000 ms; TE, 35 ms; FA, 90º; FOV, 220 × 220 mm^2^; slice thickness, 4.0 mm; number of slices, 35; and the duration of scan time, 5:09. Participants were instructed to think about nothing in particular, to keep their eyes closed, and to stay awake. An automated shimming procedure was used to reduce the influence of field inhomogeneities before performing functional MRI scans.

Functional imaging data were pre-processed using FMRIB Software Library tools (FSL, http://www.fmrib.ox.ac.uk/fsl). Standard pre-processing steps were conducted, including motion correction using multi-resolution rigid body co-registration, brain extraction using the FSL Brain Extraction Tool (BET)^35^, spatial smoothing using a Gaussian kernel (5 mm), and high-pass filtering (0.01 Hz). Functional imaging data for each individual were co-registered with the corresponding T1-weighted structural image. These co-registered images were then normalized to the Montreal Neurological Institute (MNI) atlas using affine registration (12 degrees of freedom). The pre-processed images were then concatenated in the temporal dimension to create a single four-dimensional (4D) dataset. There were no significant differences in head motion parameters of TGA patients and healthy volunteers for absolute head motion (TGA group, 0.224 ± 0.233 mm; controls, 0.161 ± 0.020; *t* = − 1.28; *P* = 0.203) or relative head motion (TGA group, 0.086 ± 0.040 mm; Controls, 0.080 ± 0.033 mm; *t* = − 0.61; *P* = 0.55).

Single-subject independent component analyses (ICA) were performed using multivariate exploratory linear optimized decomposition into independent components (MELODIC)^36^. Then, the ICA-based Xnoiseifier (FIX) of the FMRIB was used to remove components corresponding to structural artifacts from each functional image data set^37^. A group ICA, which is a model-free and data-driven approach, was performed to separate 4D functional MRI data into a set of independent one-dimensional (1D) time-series and related 3D spatial maps^36,38^ based on the unified group from the whole participants (50 TGA patients and 25 controls). In the current study, functional imaging data were decomposed into 20 independent components (dimensionality = 20) using a temporal concatenation approach. Consequently, 10 components were classified as anatomically and functionally meaningful resting state networks (RSNs) corresponding to functional networks previously described^38^, while 10 components were classified as artifacts following the visual inspection of an experienced researcher (G.H.). Of the 10 RSNs that were identified, we selected three RSNs of interest, including the SN, DMN, and CEN. The three aforementioned RSNs of interest were used in all subsequent analyses. Component information and spatial maps of the available components with a threshold of z = 3.1 *(P* = 0.001) are presented in Figure e-5. We also obtained a group ICA map with 26 hyperacute TGA patients and 25 controls, which was similar to that of whole participants (Figure e-6).

A dual linear regression approach was applied to identify subject-specific time courses and spatial maps^36^. The group ICA map for the SN, DMN and CEN based on the whole participants was used to perform linear model fit against each subject’s functional MRI and to create the average time course within the SN, DMN and CEN for each subject (spatial regression). The time courses of the subjects were variance-normalized and then regressed against the subject’s functional MRI data to create subject-specific spatial maps for the SN, DMN, and CEN (temporal regression).

### Statistical analysis

Demographic and clinical characteristics were compared between patients with TGA and controls using independent t tests for continuous variables and chi-square tests or Fisher’s exact tests for categorical variables, respectively.

Group differences in functional connectivity were analyzed using a voxel-wise t test. Voxel-wise analyses were performed using an ROI mask of the SN, DMN and CEN. The results were corrected for multiple comparisons using the Monte Carlo simulation adjusting for 10,000 iterations implemented in the AlphaSim utility (http://afni.nimh.nih.gov/pub/dist/doc/program_help/AlphaSim.html). A threshold derived from a combination of *P* < 0.01 and a cluster size of a minimum of 336 mm^3^ was used to correct for multiple comparisons at *P* < 0.05. An alpha-level of *P* < 0.05 (two-tailed) was considered statistically significant for all analyses. The Z values that represent the functional connectivity of the corresponding RSNs, were extracted from the ROI masks of the SN, DMN and CEN, which was then used for further analyses. The Z values of each three functional connectivity in TGA patients were converted to normalized z-values using the baseline mean scores and standard deviation of the controls. The normalized z-values were used to show the longitudinal changes of RSNs in TGA patients.

General linear model was performed to examine the group differences in functional connectivity of the corresponding RSNs after adjusting age and sex, while a linear mixed- effect model was employed to estimate the time effects on the RSNs in TGA patients. This model included functional connectivity of the RSNs as linear terms and within-individual variations as random effects. Age and gender were included as covariates.

Data were analyzed using Stata v13.1 (StataCorp., College Station, TX).

## Supplementary Information

Supplementary Information.

## Data Availability

Data and statistical algorithms used for analyses are available from the corresponding author on reasonable request.

## References

[1] Quinette P (2006). What does transient global amnesia really mean?-review of the literature and thorough study of 142 cases. Brain J. Neurol..

[2] Fisher CM, Adams RD (1964). Transient global amnesia. Acta Neurologica Scandinavica. Supplementum.

[3] Bartsch T, Deuschl G (2010). Transient global amnesia: functional anatomy and clinical implications. The Lancet. Neurol..

[4] Bartsch T (2006). Selective affection of hippocampal CA-1 neurons in patients with transient global amnesia without long-term sequelae. Brain J. Neurol..

[5] Bartsch T, Alfke K, Deuschl G, Jansen O (2007). Evolution of hippocampal CA-1 diffusion lesions in transient global amnesia. Ann. Neurol..

[6] Sedlaczek O (2004). Detection of delayed focal MR changes in the lateral hippocampus in transient global amnesia. Neurology.

[7] Fransson P (2005). Spontaneous low-frequency BOLD signal fluctuations: an fMRI investigation of the resting-state default mode of brain function hypothesis. Hum. Brain Mapp.

[8] Bonnelle V (2012). Salience network integrity predicts default mode network function after traumatic brain injury. Proc. Natl. Acad. Sci. U S A.

[9] Menon V, Uddin LQ (2010). Saliency, switching, attention and control: a network model of insula function. Brain Struct. Funct..

[10] Jilka SR (2014). Damage to the salience network and interactions with the default mode network. J. Neurosci..

[11] Menon V (2011). Large-scale brain networks and psychopathology: a unifying triple network model. Trends Cogn. Sci..

[12] Seeley WW (2007). Dissociable intrinsic connectivity networks for salience processing and executive control. J. Neurosci..

[13] Gusnard DA, Akbudak E, Shulman GL, Raichle ME (2001). Medial prefrontal cortex and self-referential mental activity: relation to a default mode of brain function. Proc. Natl. Acad. Sci. U S A.

[14] Buckner RL, Andrews-Hanna JR, Schacter DL (2008). The brain's default network: anatomy, function, and relevance to disease. Ann. N. Y. Acad. Sci..

[15] Park YH (2014). Disruption of the posterior medial network during the acute stage of transient global amnesia: a preliminary study. Clin. EEG Neurosci..

[16] Peer M (2014). Reversible functional connectivity disturbances during transient global amnesia. Ann. Neurol..

[17] Zidda F (2019). Resting-state connectivity alterations during transient global amnesia. NeuroImage. Clin..

[18] Quinette P (2006). What does transient global amnesia really mean? Review of the literature and thorough study of 142 cases. Brain J. Neurol..

[19] Sridharan D, Levitin DJ, Menon V (2008). A critical role for the right fronto-insular cortex in switching between central-executive and default-mode networks. Proc. Natl. Acad. Sci..

[20] Qin P, Northoff G (2011). How is our self related to midline regions and the default-mode network?. Neuroimage.

[21] Ranganath C, Ritchey M (2012). Two cortical systems for memory-guided behaviour. Nat. Rev. Neurosci..

[22] Bartsch T, Butler C (2013). Transient amnesic syndromes. Nat. Rev. Neurol..

[23] Philippi CL, Tranel D, Duff M, Rudrauf D (2015). Damage to the default mode network disrupts autobiographical memory retrieval. Soc. Cogn. Affect. Neurosci..

[24] Hodges JR (1994). Semantic memory and frontal executive function during transient global amnesia. J. Neurol Neurosurg. Psychiatry.

[25] Quinette P (2003). Working memory and executive functions in transient global amnesia. Brain J. Neurol..

[26] Banich MT (2009). Cognitive control mechanisms, emotion and memory: a neural perspective with implications for psychopathology. Neurosci. Biobehav. Rev..

[27] Jang JW (2015). Longitudinal cerebral perfusion change in transient global amnesia related to left posterior medial network disruption. PLoS ONE.

[28] Wang X, Zhang W, Sun Y, Hu M, Chen A (2016). Aberrant intra-salience network dynamic functional connectivity impairs large-scale network interactions in Schizophrenia. Neuropsychologia.

[29] Pannekoek JN (2013). Aberrant limbic and salience network resting-state functional connectivity in panic disorder without comorbidity. J. Affect. Disord..

[30] Zhang W (2019). Acute stress alters the ‘default’ brain processing. Neuroimage.

[31] Hodges J, Warlow C (1990). Syndromes of transient amnesia: towards a classification. A study of 153 cases. J. Neurol. Neurosurg. Psychiatry.

[32] Caplan, L. *Transient Global Amnesia Handbook of Clinical Neurology*. Vol. 45 (1998).

[33] Han C (2008). An adaptation of the Korean mini-mental state examination (K-MMSE) in elderly Koreans: demographic influence and population-based norms (the AGE study). Arch. Gerontol. Geriatr..

[34] Ahn HJ (2010). Seoul Neuropsychological Screening Battery-dementia version (SNSB-D): a useful tool for assessing and monitoring cognitive impairments in dementia patients. J. Korean Med. Sci..

[35] Jenkinson M, Bannister P, Brady M, Smith S (2002). Improved optimization for the robust and accurate linear registration and motion correction of brain images. Neuroimage.

[36] Beckmann, C. F., DeLuca, M., Devlin, J. T. & Smith, S. M. Investigations into resting-state connectivity using independent component analysis. *Philos. Trans. R. Soc. London. Ser. B Biol. Sci.* **360**, 1001–1013. 10.1098/rstb.2005.1634 (2005). 10.1098/rstb.2005.1634 PMC1854918 16087444

[37] Griffanti L (2014). ICA-based artefact removal and accelerated fMRI acquisition for improved resting state network imaging. Neuroimage.

[38] Smith SM (2009). Correspondence of the brain's functional architecture during activation and rest. Proc. Natl. Acad. Sci. U S A.

