# Supplementary material for: Aberrantly higher functional connectivity in the salience network is associated with transient global amnesia
Source: Sci Rep. 2021 Oct 18;11:20598. doi: 10.1038/s41598-021-97842-y (PMC8523536; doi:10.1038/s41598-021-97842-y)
Supplement: Supplementary file 1 — Supplementary Information. [file 41598_2021_97842_MOESM1_ESM.pdf]

Table e-1. Demographic characteristics of TGA patients who did and did not take part in the 3-month follow-up visit

Mean ± SD	**3-month FU** (n = 22)	**Non-FU** (n = 28)	P-value
Age (year)	59.7 ± 5.3	61.4 ± 6.9	0.35
Education	10.5 ± 2.7	11.7 ± 4.2	0.23
Gender (% of female)	19 (86.4)	23 (82.1)	0.49
Risk factors			
Hypertension, n (%)	6 (27.3)	8 (28.6)	0.59
Diabetes Mellitus, n (%)	1 (4.6)	2 (66.7)	0.59
Hyperlipidemia, n (%)	4 (18.2)	6 (21.4)	0.53
Cardiac disease, n (%)	2(9.1)	2(7.1)	0.60
Stroke, n (%)	1 (4.6)	2 (7.1)	0.59
Migraine, n (%)	2 (9.1)	5 (17.9)	0.32
TGA symptoms			
Duration of TGA symptoms (hr)	9.4 (11.3)	10.3 (9.5)	0.77
K-MMSE (during ER visit)	23.0 ± 2.7	23.3 ± 3.4	0.82
K-MMSE (72 hours)	28.1 ± 2.0	27.7 ± 1.6	0.45
Hyperintense lesions on DWI, n (%)	18 (52.9)	16 (47.1)	0.08

Abbreviations: SD, standard deviation; FU, took part in the follow-up visit; Non-FU, did not take part in the follow-up visit; K-MMSE, Korean version of mini-mental state examination; min, minutes; DWI, Diffusion weighted imaging

Figures e-1. Longitudinal changes of topographical functional networks in TGA patients

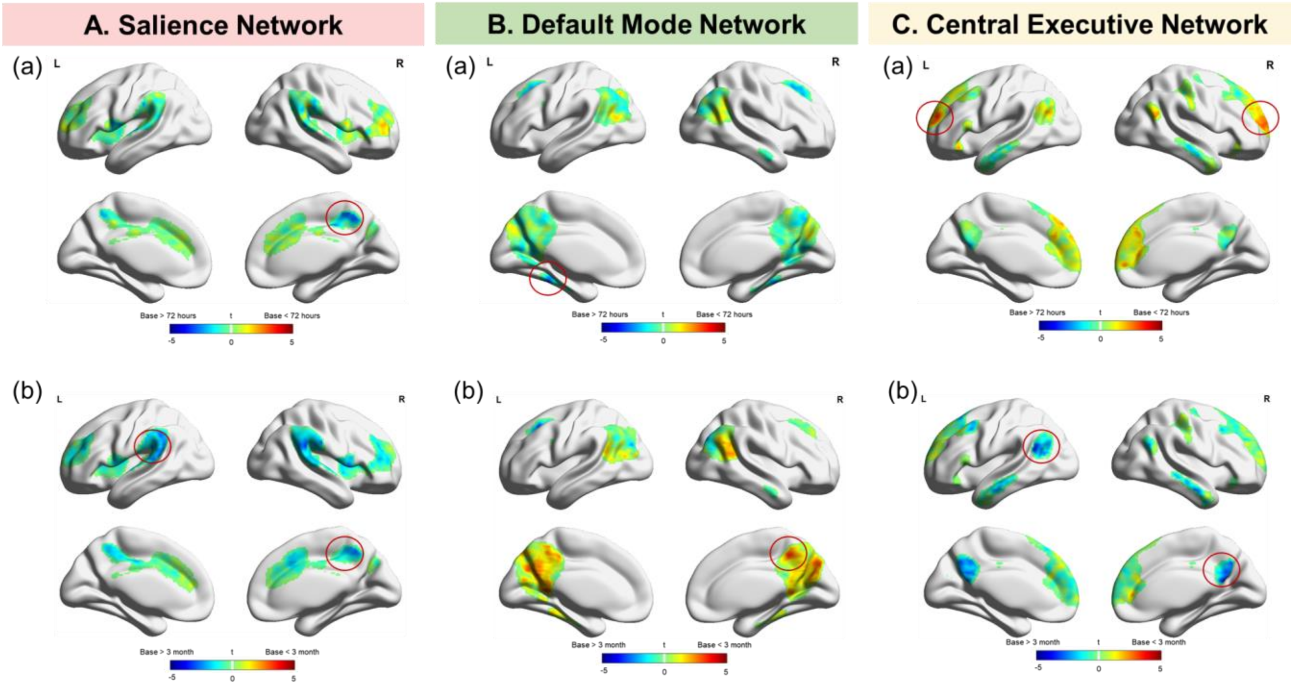

* Red circle represents significant clusters indicating differences of functional connectivity compared to the baseline in TGA patients.

A.(a) T-static maps of functional connectivity on the SN in 72 hours. Compared to the baseline, TGA patients showed decreased functional connectivity on the right precuneus within 72 hours
(b) T-static maps of functional connectivity on the SN in 3 months. Compared to the baseline, TGA patients demonstrated decreased functional connectivity on the left temporo-parietal junction and right precunes in 3 months
B.(a) T-static maps of functional connectivity on the DMN in 72 hours. Compared to the baseline, TGA patients illustrated decreased functional connectivity on the left parahippocampal gyrus within 72 hours
(b) T-static maps of functional connectivity on the DMN in 3 months. Compared to the baseline, increased functional connectivity on the right precunes was observed in 3 months.
C.(a) T-static maps of functional connectivity on the CEN in 72 hours. Compared to the baseline, decreased functional connectivity on the left parahippocampal gyrus was noted within 72 hours
(b) T-static maps of functional connectivity on the CEN in 3 months. Compared to the baseline, decreased functional connectivity on the right precunes and left temporo-parietal junction was shown in 3 months.

Figures e-2. Comparison of functional brain network during acute phase between the hyperacute and post-acute TGA patients.

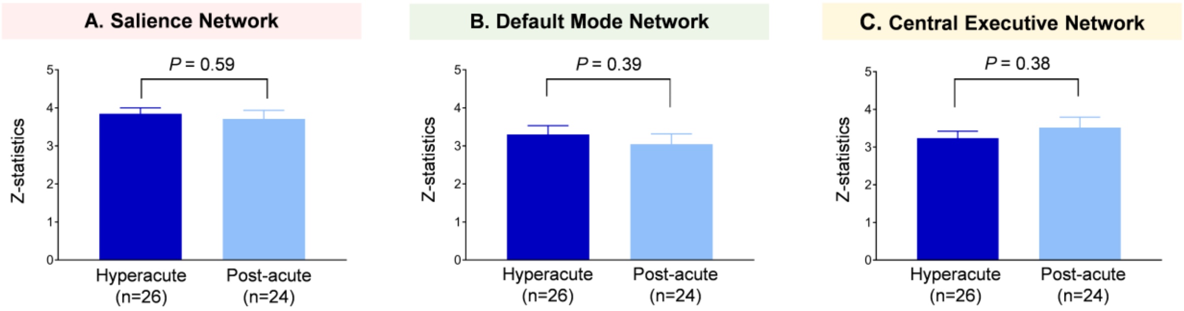

* *P* value was adjusted by age and sex.

There was no statistical difference of the functional connectivity on the SN, DMN and CEN between the hyperacute and post-acute TGA patients.

Figures e-3. Comparison of functional connectivity between TGA patients with hippocampal lesion and those without hippocampal lesion on DWI

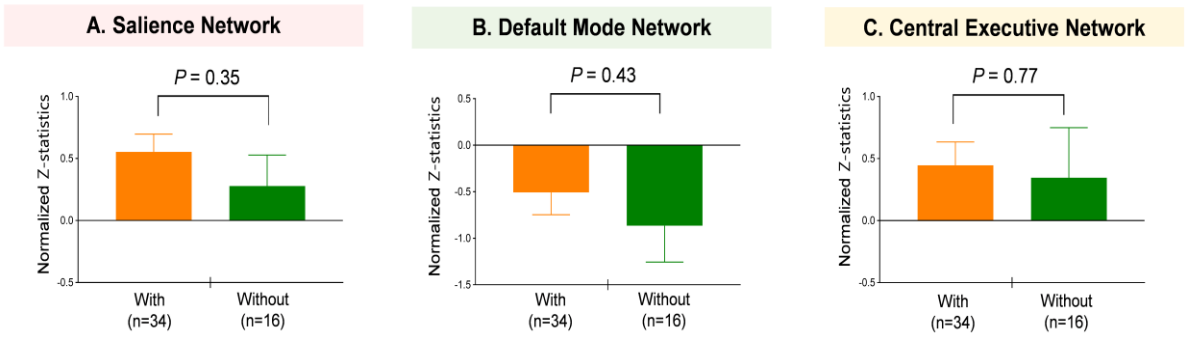

* *P* value was adjusted by age and sex.

There were no significant differences of global functional connectivity in the SN, DMN and CEN between TGA patients with hippocampal lesion (n=34) and those without hippocampal lesion (n=16)

Figures e-4. Comparison of functional connectivity between TGA patients with emotional cause and those with other causes

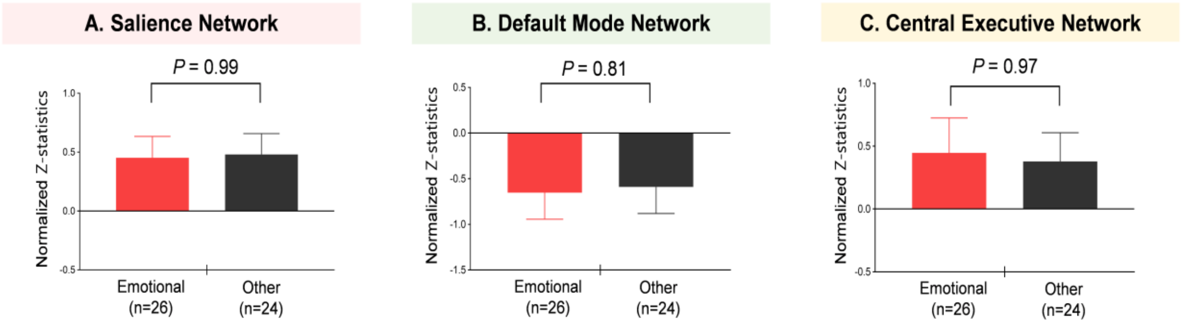

* *P* value was adjusted by age and sex

To investigate the effects of precipitating emotional causes on intrinsic brain networks, we compared functional connectivity of the SN, DMN and CEN between TGA patients with emotional causes (n=26) and those with other causes (n=24). However there were no statistical differences of functional connectivity between the two groups

Figures e-5. Large scale functional brain network identified using a group independent component analysis (ICA).

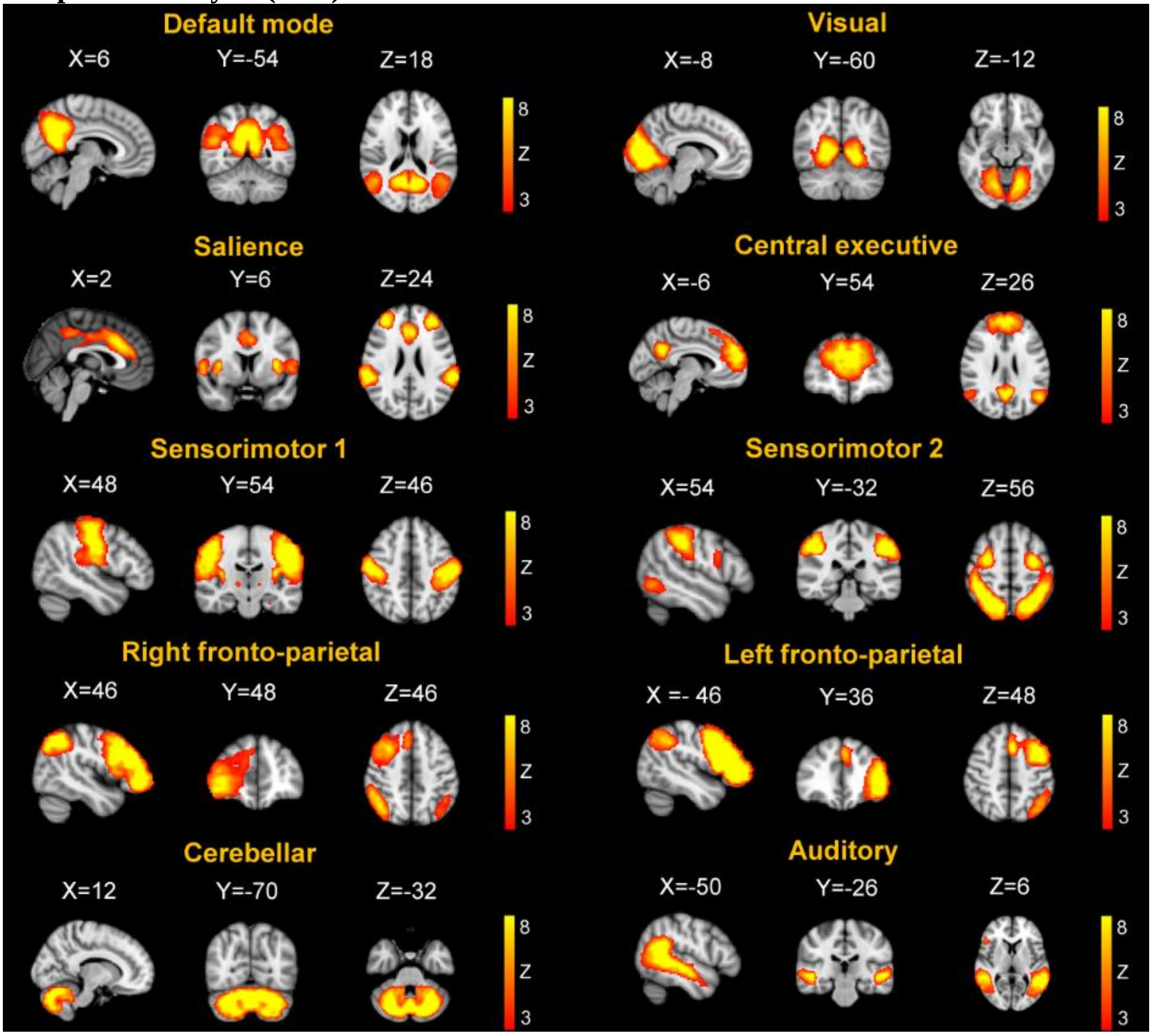

A group ICA with TGA patients (n=50) and controls (n=25) identified 10 independent components, which corresponded to classic resting state networks. Spatial maps were converted to Z score images and were thresholded at Z = 3.1 (P = 0.001).

Figures e-6. Large scale functional brain network identified using a group independent component analysis (ICA) in matched participants

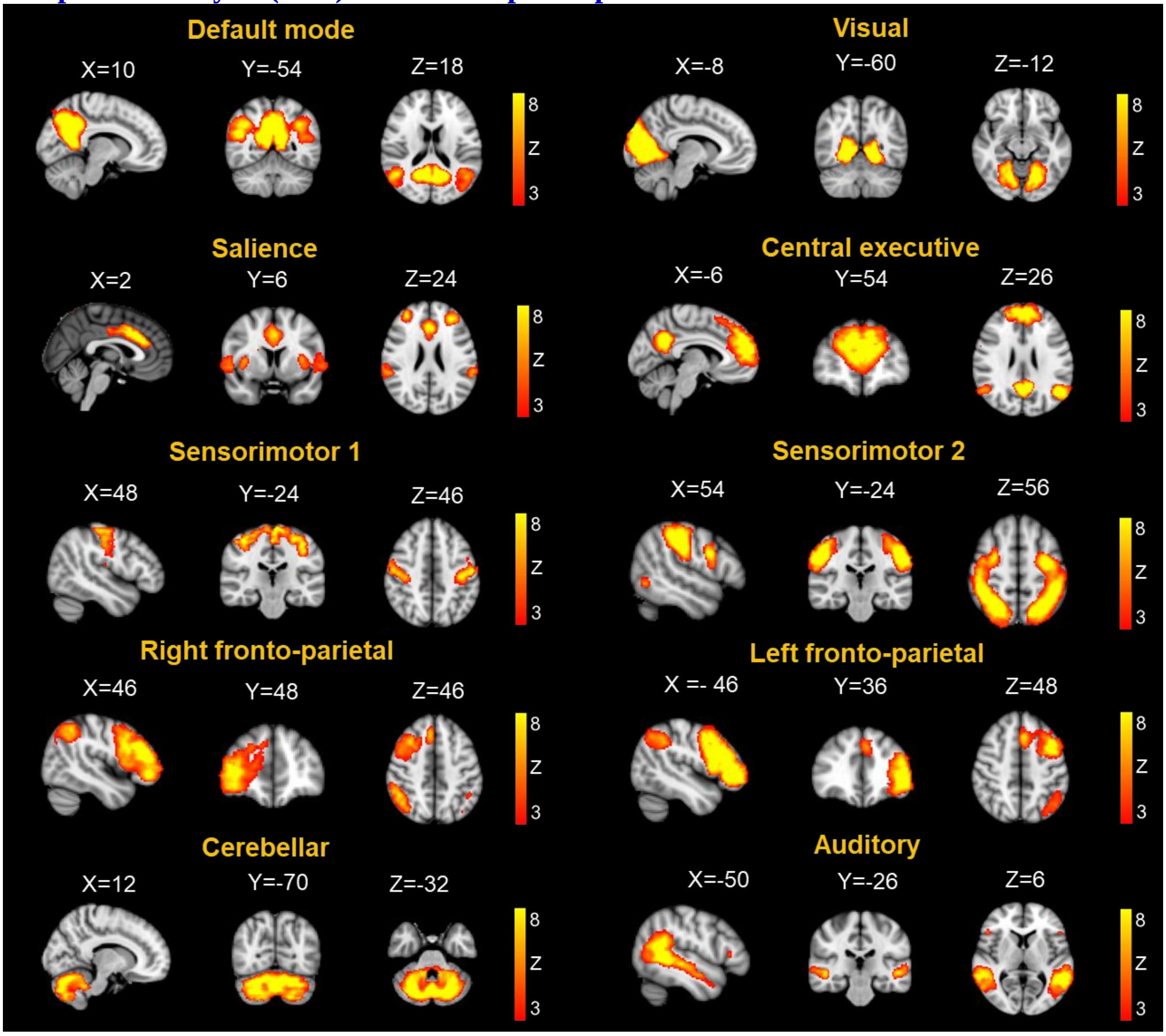

A group ICA with a comparable number of TGA patients (n= 26 in hyperacute phase) and controls (n= 25) identified 10 independent components, which corresponded to classic resting state networks. Spatial maps were converted to Z score images and were thresholded at Z = 3.1 (P = 0.001).
